# Exploration of the Effects of Different *Beauveria bassiana* Strains on *Dioryctria sylvestrella* Larvae from the Perspective of Oxidative Stress

**DOI:** 10.3390/insects16060640

**Published:** 2025-06-18

**Authors:** Ruting Chen, Meiling Wang, Hanwen Zhang, Jianjiao Xu, Xiaomei Wang, Defu Chi, Jia Yu

**Affiliations:** 1 Key Laboratory for Sustainable Forest Ecosystem Management of Ministry of Education, College of Forestry, Northeast Forestry University, Harbin 150040, China; crt1391403031@126.com (R.C.);; 2 Fuyuan City Forestry and Grassland Development Center, Fuyuan Forestry and Grassland Bureau, Jiamusi 156500, China; 3 Fuyuan Forest Farm, Jiamusi 156500, China

**Keywords:** *Dioryctria sylvestrella*, *Beauveria bassiana*, oxidative stress response, immune defense, infection process

## Abstract

(1) Background: The larvae of *Dioryctria sylvestrella* typically bore into the shoots and cones of *Pinus koraiensis*, increasing tree breakage risk and reducing cone yield. (2) Methods: Five *Beauveria bassiana* strains were evaluated for virulence against fourth-instar larvae. And the levels of T-AOC and MDA in the larvae infected by each strain were measured. To assess larval responses to different strains, we measured the activities of six enzymes (SOD, CAT, POD, PPO, CarE, GST) and the levels of GSH and H_2_O_2_ in larvae treated with each strain. Additionally, the infection process of highly pathogenic *B. bassiana* in larvae was explored using scanning electron microscopy (SEM). (3) Results: Strain CGMCC3.2055 demonstrated the highest toxicity to larvae, achieving a cumulative corrected mortality of 80.56% on the 4th day and an LT_50_ of 3.248 days. The T-AOC of larvae treated with strain CGMCC3.2055 was inhibited within 48 h. The relative MDA content in this group was significantly higher than that in other strain-treated groups at 6, 12, and 24 h. In Bb01-treated larvae, H_2_O_2_ accumulation at 6 and 24 h post-infection was influenced by POD activity rather than GSH levels; in BbZ1-treated larvae, the activities of CAT and POD were upregulated at 6 and 36 h, while the activity of SOD was downregulated, but the content of H_2_O_2_ increased significantly, resulting in accumulation; in CFCC81428-treated larvae, a decline in T-AOC coincided with substantial H_2_O_2_ accumulation over 48 h, while a concomitant increase in GSH content bolstered tolerance to lethal oxidative damage; in CGMCC3.2055-treated larvae, H_2_O_2_ only accumulated significantly at 24 and 48 h, yet upregulated CAT and POD were insufficient to effectively scavenge the excess H_2_O_2_; and in bio-21738-treated larvae, SOD-driven dismutation generated substantial H_2_O_2_ from 12 to 48 h, leading to pronounced accumulation from 6 to 48 h, yet limited upregulation of POD (only at 6 and 12 h) and CAT (only at 12 and 48 h) were insufficient to mitigate H_2_O_2_ buildup. PPO activity was upregulated within 48 h in all treatment groups except for BbZ1, where no upregulation was observed at 12 and 48 h. GST activity was upregulated in all treatment groups except for CGMCC3.2055, where a downregulation was observed at 12 h post-infection. CarE activity was significantly upregulated within 48 h in both CFCC81428 and CGMCC3.2055 groups; in the Bb01 group, CarE was upregulated only at 6 and 48 h; in the BbZ1 group, CarE was downregulated only at 48 h; and in the bio-21738 group, CarE showed no upregulation at 24 and 48 h. Through SEM, the infection process of the strain CGMCC3.2055 on the surface of the larvae was further determined, which mainly included adhesion, the appearance of bud-like protrusions, the growth of germ tubes along the epidermis and penetration of the epidermis, as well as the colonization of the strain and its emergence from the surface of the larvae. (4) Conclusions: This study first screened the highly pathogenic *B. bassiana* strain CGMCC3.2055 by evaluating its virulence to larvae and post-infection T-AOC and MDA levels. It also clarified the strain’s infection process and the larvae’s immune responses to various strains.

## Simple Summary

The larvae of Dioryctria sylvestrella typically bore into the whorled branches of main shoots, apical shoots, and cones of Pinus koraiensis, increasing tree breakage risk and reducing cone yield. Currently, the control of such pests mainly relies on chemical insecticides, but the extensive application of these chemicals is highly likely to lead to pest resistance and environmental pollution. In order to effectively control the damage caused by D. sylvestrella and reduce the environmental risks posed by insecticides, this study aims to screen entomopathogenic fungi that are highly pathogenic to the larvae of D. sylvestrella as a substitute for the use of traditional insecticides. For the first time, we screened the highly pathogenic strain CGMCC3.2055 by evaluating the virulence of B. bassiana against D. sylvestrella larvae, along with their T-AOC and MDA levels post-infection. We further investigated the larvae’s immune responses to different strains and elucidated the infection process of CGMCC3.2055, laying a theoretical foundation for using B. bassiana to control D. sylvestrella and exploring the strain’s pathogenic mechanism.

## 1. Introduction

*Pinus koraiensis*, a national second-class protected plant, is an indigenous tree species in Northeast China, with its trunk, branches, and bark utilized for timber and its pinenut kernels highly valued for edible purposes [1]. In recent years, the increasing area of *P. koraiensis* plantations in Northeast China, with its single-species cultivation, has triggered the outbreak of insect pests mainly represented by *Dioryctria sylvestrella*, whose larvae frequently damage the whorled branches of the main shoots, apical shoots, and cones of the trees [2]. The forest trees that have been damaged will have an increased risk of breakage due to the feeding behavior of the larvae [3], resulting in a reduction in the yield of cones in a large number of *P. koraiensis* plantations. Investigations have found that in the Jiamusi area, the damage rates of *P. koraiensis* cones and seeds caused by *D. sylvestrella* have reached over 98% and 56%, respectively [4]. Currently, the prevention and control of this pest mainly rely on chemical control. However, the long-term and improper use of pesticides will give rise to severe pesticide residue issues. Additionally, the extensive application of pesticides can readily increase the pests’ resistance [5]. Biological control, as a key component of the Integrated Pest Management (IPM) program, has effectively addressed this issue [6].

*Beauveria bassiana* serves as a biocontrol strain for managing multiple pests, attributed to its wide host range. *B. bassiana* usually infects hosts by penetrating the insect cuticle through contact, and eventually colonizes within the host, leading to the death of the insect [7]. However, the pathogenicity of *B. bassiana* varies among different insect hosts. Some research has indicated that when *B. bassiana* infects non-initial hosts, its strains will undergo changes in metabolic responses and infection strategies to adapt to the new hosts [8]. However, the differences in the pathogenicity of different strains of *B. bassiana* to the same insect host under the same conditions are largely attributed to the unique gene expression patterns that commonly occur during the early stage of the strains’ infection of the insect host [9]. Sihyeon Kim et al. [10] used transcriptomics to study the infection patterns of the cuticles of *Frankniella occidentalis* by two strains of *B. bassiana* with similar morphology, virulence, and genomic structures, and found that there were differences in the initial infection steps and the interactions with the cuticles of *F. occidentalis* were not identical. When insects are faced with the infection of *B. bassiana*, their internal immune systems will generate a high level of reactive oxygen species (ROS) to neutralize the infection of *B. bassiana*, but an excessive amount of ROS will cause an oxidative stress response in their bodies and produce a large amount of toxic hydrogen peroxide (H_2_O_2_), which will disrupt the antioxidant–prooxidant homeostasis within the insect cells, ultimately leading to the death of insects due to oxidative damage, immunotoxicity, and apoptosis [11]. However, the total antioxidant capacity (T-AOC) and the content of malondialdehyde (MDA) in insects can effectively reflect the overall antioxidant level of the insect body and the degree of damage to lipids caused by peroxidation [12]. The research has found that the oxidative stress mechanism mainly balances the level of ROS in cells by regulating the activity of antioxidant enzymes and the level of antioxidant substances, among which the antioxidant enzymes and antioxidant substances mainly include superoxide dismutase (SOD), catalase (CAT), peroxidase (POD), reduced glutathione (GSH), etc. [13]. However, apart from the oxidative stress response in the defensive reaction of insects to *B. bassiana*, the detoxifying enzymes and polyphenol oxidase (PPO) in their bodies also resist the infection of *B. bassiana* in different ways, for example, the carboxylesterase (CarE) in insects can participate in the detoxification of exogenous substances by hydrolyzing the carboxylester bond [14]. While PPO can resist the infection of *B. bassiana* through the melanization reaction [15].

Therefore, in this study, for the first time, one strain with high pathogenicity to the larvae of *D. sylvestrella* was screened out in terms of three aspects, namely the pesticidal effect of *B. bassiana* on the larvae of *D. sylvestrella*, the total antioxidant capacity of the larvae after being infected by the strain, and the degree of damage to lipids caused by peroxidation. We then determined the infection process of this strain on the larvae and explored the immune defense responses of the larvae to different strains of *B. bassiana*. The study provides a theoretical basis for the subsequent biological control of the larvae of *D. sylvestrella*, as well as for the in-depth exploration of the interaction mechanism between *B. bassiana* and *D. sylvestrella*.

## 2. Materials and Methods

Test insects: The larvae of *D. sylvestrella* were sourced from the damaged shoots within the artificial plantation of *P. koraiensis* in Boli County, Qitaihe City, Heilongjiang Province (45°45′36″ N, 130°23′56″ E).

Test strains: In this experiment, five strains of *B. bassiana* were selected. They are Bb01 and CFCC81428, which are highly pathogenic to the larvae of *D. abietella* [16,17]; BbZ1, which is highly pathogenic to the adults of *Hylurgus ligniperda* [18]; and CGMCC3.2055 and bio-21738, which were isolated from the bodies of *Dendrolimus* SPP. *B. bassiana* strains Bb01 and BbZ1 were isolated in the laboratory. *B. bassiana* CFCC81428 was provided by Beijing Beina Chuanglian Biotechnology Research Institute (BNCC, Beijing, China), *B. bassiana* CGMCC3.2055 was provided by the Beijing Collection of Genomic Microbial Culture (BJMCC, Beijing, China), and *B. bassiana* bio-21738 was provided by Beijing Biobw Biotechnology Co., Ltd. (Biobw, Beijing, China). The details of the specific strains are shown in Table 1.

Toxicity determination: The toxicity determination to the larvae of *D. sylvestrella* was carried out with slight modifications based on the immersion method described by Zhang et al. [11]. Five strains of *B. bassiana* were inoculated onto SDAY solid medium and incubated in the dark in an artificial incubator at 25 °C and 90% relative humidity (RH) for 15 days to obtain a large number of conidia. The conidia were scraped off into a 1.5 mL sterile centrifuge tube and stored at 4 °C for future use. A certain amount of conidia was taken and placed into a new 1.5 mL sterile centrifuge tube; then, 1 mL of sterile 0.5% Tween-80 was added and fully shaken. The concentration of the conidia were determined using the hemocytometer method, and the conidia were diluted with sterile 0.5% Tween-80 to prepare a conidial suspension at a concentration of 1.0 × 10^7^ conidia/mL. The 4th-instar larvae of *D. sylvestrella* were immersed in 20 mL of the conidial suspension for 30 s, then placed into a 24-well plate lined with sterile filter paper, and the same volume of sterile artificial diet was added to the plate. The control group (CK) consisted of larvae immersed in 0.5% Tween-80, with the rest of the operations being the same as those for the treatment group. Three replicates were set up in total, with 12 test insects in each replicate. The test insects in the treatment and CK groups were cultured in an artificial incubator at 25 °C and 90% RH, and the number of dead test insects was recorded every 24 h for a total of 7 days. Dead test insects were placed in a 24-well plate lined with sterile moist filter paper, and the growth of fungi on the insect bodies indicated that they were killed by *B. bassiana*.

Determination of the antioxidant and detoxification systems in larvae: The 4th-instar larvae of *D. sylvestrella* were infected with the strains using the same method as the toxicity determination. High-vitality larvae infected with each strain for 6 h, 12 h, 24 h, 36 h, and 48 h were selected, quickly frozen in liquid nitrogen, and then stored in a −80 °C freezer. The activities of T-AOC, SOD, CAT, POD, PPO, CarE, and glutathione S-transferase (GST), as well as the contents of H_2_O_2_, GSH, and MDA in the larvae infected with each strain for different durations were determined. All the kits used for the determination were purchased from Suzhou Grace Biotechnology Co., Ltd. (Suzhou, China). For sample detection, 0.1 g of *D. sylvestrella* larvae was taken for sample preparation according to the instructions of the kit. The measurement was carried out on a microplate reader (FlexA-200 Full Wavelength Microplate Reader, Hangzhou Aosheng Instrument Co., Ltd., Hangzhou, China). Each treatment was repeated three times.

Scanning electron microscopy (SEM): The treatment method for the 4th-instar larvae of *D. sylvestrella* was the same as that for the toxicity determination. After the larvae were immersed in the spore suspension of the screened highly pathogenic strain with a concentration of 1.0 × 10^7^ conidia/mL for 30 s, samples were taken at 8, 16, 24, 48, 72, and 84 h after the treatment, respectively. The samples were placed in a 2.5% glutaraldehyde fixative solution, wrapped in tin foil, and fixed overnight for 24 h. The samples were rinsed three times with phosphate buffer (0.1 mol/L, pH = 7.4; 15 min for each rinse). Gradient dehydration was carried out using 30%, 50%, 70%, 80%, 90%, and 100% ethanol, with each step lasting 30 min. After dehydration, the samples were dried in an electric blast dryer at 40 °C for 6 h and placed on the sample holder with conductive adhesive according to different observation surfaces. Finally, ion sputtering for gold coating was performed for 2 min. The infected larval samples were photographed using a SEM (HITACHI S-3400N, Tokyo, Japan).

Data processing: Data calculations were performed using Excel 2016, with the cumulative corrected mortality calculated as [(cumulative mortality of the treatment group−cumulative mortality of the control group)/(1−cumulative mortality of the control group)] × 100% [16]; one-way analysis of variance (ANOVA) was conducted using IBM SPSS Statistics 27 to test the significance of differences among different treatments via Duncan’s multiple comparison method at a significance level of 0.05, while the natural logarithm transformation of the number of dead insects was carried out through the probit analysis method to calculate the toxicity regression equations and median lethal time (LT_50_) of each strain against the larvae. The circular bar charts were created using R version 4.5.0 within the RStudio Desktop environment, and the circlize and readxl packages were mainly applied.

## 3. Results

### 3.1. Screening of Highly Pathogenic Strains

**Figure 1:**
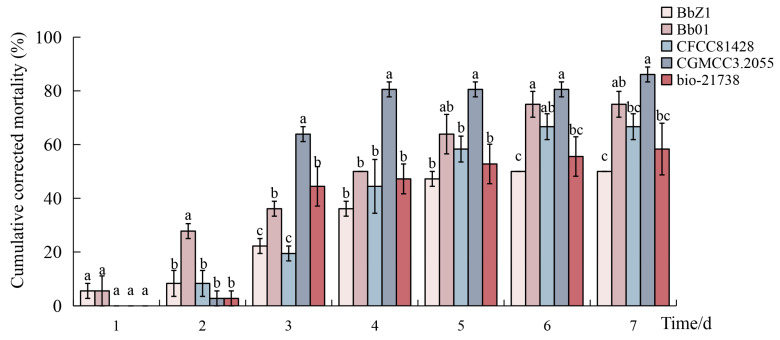
The cumulative corrected mortality of *D. sylvestrella* larvae induced by different *B. bassiana* strains. Different lowercase letters indicate statistically significant differences in the cumulative corrected mortality among different strains at the 0.05 level within the same time period (Duncan’s test, *p* < 0.05).

At a concentration of 1.0 × 10^7^ conidia/mL, the cumulative corrected mortalities of the fourth-instar larvae of *D. sylvestrella* caused by five *B. bassiana* strains (BbZ1, Bb01, CFCC81428, CGMCC3.2055, and bio-21738) are shown in Figure 1. On the 2nd day after the larvae were infected by *B. bassiana*, the cumulative corrected mortality of the larvae treated with strain Bb01 was significantly higher than that of the other strains (*p* < 0.05). On the 3rd day after infection, the cumulative corrected mortality of the larvae treated with strain CGMCC3.2055 was 63.89 ± 2.78%, significantly higher than that of the other strains (*p* < 0.05), while the cumulative corrected mortalities of the larvae treated with strains Bb01 and bio-21738, ranking second only to strain CGMCC3.2055, were significantly higher than those of strains BbZ1 and CFCC81428 (*p* < 0.05). On the 4th day after infection, the cumulative corrected mortality of the larvae treated with strain CGMCC3.2055 was the highest, reaching 80.56 ± 2.78%. On the 7th day after the five *B. bassiana* strains infected the larvae of *D. sylvestrella*, only the cumulative corrected mortality of the larvae treated with strain CGMCC3.2055 exceeded 80%.

The virulence regression equations and LT_50_ of the five *B. bassiana* strains against the larvae are shown in Table 2. Among them, the LT_50_ values of strains CGMCC3.2055 and Bb01 are both less than 4 days, which are 3.248 days and 3.739 days, respectively. Combining with the cumulative corrected mortality of the larvae, it can be determined that strain CGMCC3.2055 has high pathogenicity to the larvae, and the pathogenic effect of strain Bb01 is second only to that of strain CGMCC3.2055.

### 3.2. The Defense Response System of D. sylvestrella Larvae Against B. bassiana

#### 3.2.1. The Influence of *B. bassiana* on the Antioxidant Capacity of *D. sylvestrella* Larvae

**Figure 2:**
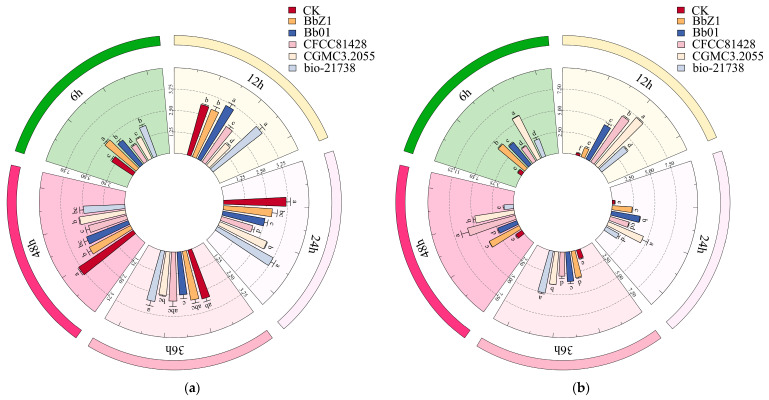
Effects of different *B. bassiana* strains on the antioxidant capacity of *D. sylvestrella* larvae. (**a**) T-AOC relative activity (μmol Trolox/g); (**b**) MDA relative content (nmol/g). Different lowercase letters indicate the statistical differences at the 0.05 significance level (Duncan’s test, *p* < 0.05) in the relevant activities or contents of the larvae of *D. sylvestrella* at the same time after being treated with different strains.

In this experiment, by measuring the activity of T-AOC (Figure 2a) and the content of MDA (Figure 2b) in the larvae of *D. sylvestrella* infected by five *B. bassiana* strains, the T-AOC and the degree of lipid peroxidation of the larvae against different strains were explored, and then the effects of different strains on the antioxidant capacity of the larvae were analyzed.

At 6 h after the larvae were infected, the T-AOC activities of the larvae in the treatment groups of strains BbZ1, Bb01, and bio-21738 were all significantly higher than that of the CK group (*p* < 0.05). Among them, there was no significant difference in the T-AOC activity between the CGMCC3.2055 treatment group and the CK group (*p* > 0.05). The MDA content of larvae in the CGMCC3.2055 treatment group was significantly higher than that in the other treatment groups (*p* < 0.05).

At 12 h after larval infection, the T-AOC activities of larvae in the Bb01 and bio-21738 treatment groups were significantly higher than those of the CK group and other treatment groups (*p* < 0.05), while that in the CGMCC3.2055 treatment group was significantly lower than that of the CK group and other treatment groups (*p* < 0.05), and the activity of larvae in the BbZ1 treatment group was significantly higher than that of the CFCC81428 and CGMCC3.2055 treatment groups (*p* < 0.05). The MDA contents of the larvae in all strain treatment groups were significantly higher than that of the CK group, with significant differences among the treatment groups (*p* < 0.05), and the contents ranked from high to low as strain CGMCC3.2055, CFCC81428, Bb01, bio-21738, and BbZ1.

At 24 h after the larvae were infected, only the T-AOC activity of the larvae in the bio-21738 treatment group showed no significant difference from that of the CK group (*p* > 0.05) but it was significantly higher than those of the other treatment groups (*p* < 0.05), while the activity of the larvae in the CFCC81428 treatment group was significantly lower than those of the remaining treatment groups (*p* < 0.05); there was no significant difference in the activity of the larvae between the BbZ1 treatment group and the CGMCC3.2055 and Bb01 treatment groups (*p* > 0.05), yet the activity of the larvae in the CGMCC3.2055 treatment group was significantly higher than that in the Bb01 treatment group (*p* < 0.05). The MDA content of larvae in the CGMCC3.2055 treatment group was significantly higher than that in the other treatment groups (*p* < 0.05).

At 36 h post-infection, there was no significant difference in larval T-AOC activity among the treatment groups of strains BbZ1, CFCC81428, bio-21738 and the CK group (*p* > 0.05), while the activity of larvae in the CGMCC3.2055 treatment group was significantly lower than that in the bio-21738 treatment group (*p* < 0.05). The MDA content of larvae in the CGMCC3.2055 treatment group was second only to that in the bio-21738 treatment group and significantly higher than that in the other treatment groups (*p* < 0.05).

At 48 h post-infection, the T-AOC activities of larvae in all strain treatment groups were significantly lower than that of the CK group (*p* < 0.05), and the activity of larvae in the CFCC81428 treatment group was significantly lower than those in the BbZ1 and CGMCC3.2055 treatment groups (*p* < 0.05).

#### 3.2.2. Influence of *B. bassiana* on Redox System of *D. sylvestrella* Larvae

In this experiment, the activities of SOD, CAT, POD and PPO, as well as the contents of GSH and H_2_O_2_, in the larvae of *D. sylvestrella* infected by *B. bassiana* were determined, so as to explore the effects of different strains on the redox system within the larvae of *D. sylvestrella*.

**Figure 3:**
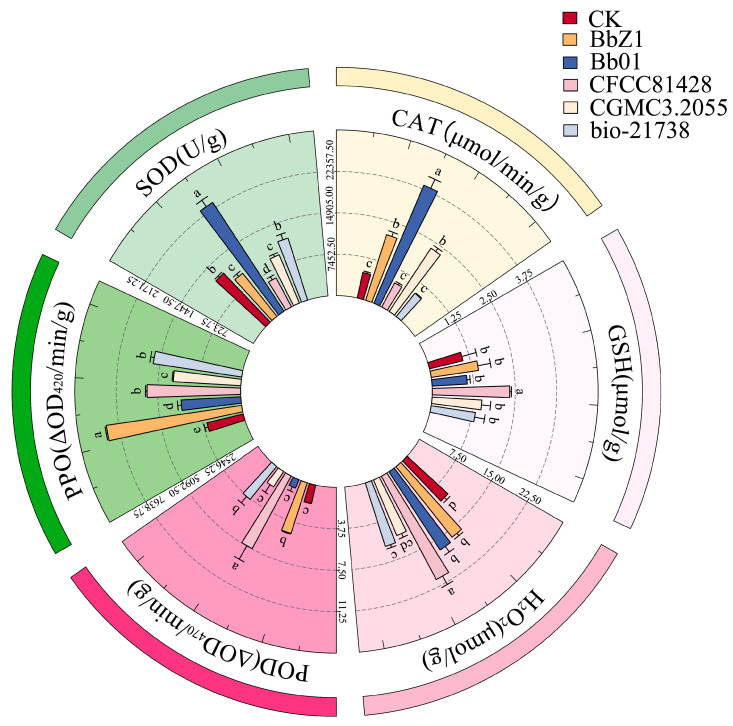
Effects of different *B. bassiana* strains on the main redox system of *D. sylvestrella* larvae at 6 h. Different lowercase letters indicate the statistical differences at the 0.05 significance level (Duncan’s test, *p* < 0.05) in the relevant activities or contents of the larvae of *D. sylvestrella* after treatment with different strains.

At 6 h post-infection (Figure 3), the SOD activity of larvae in the Bb01 treatment group was significantly higher than that of the CK group (*p* < 0.05), the CFCC81428 treatment group showed significantly lower activity than the other treatment groups (*p* < 0.05), and the activities of larvae in the BbZ1 and CGMCC3.2055 treatment groups had no significant difference (*p* > 0.05) but were significantly lower than those in the Bb01 and bio-21738 treatment groups (*p* < 0.05). The CAT activity of larvae in the Bb01 treatment group was significantly higher than that in other treatment groups (*p* < 0.05), while there was no significant difference in larval activity between the BbZ1 and CGMCC3.2055 treatment groups, which were both significantly higher than those in the CK group, as well as the CFCC81428 and bio-21738 treatment groups (*p* < 0.05). There was no significant difference in the POD activity of larvae among the treatment groups of strains Bb01 and CGMCC3.2055 and the CK group (*p* > 0.05), but their POD activities were significantly lower than those of the remaining strain treatment groups (*p* < 0.05). As an antioxidant, GSH levels in larvae 6 h post-infection were significantly higher in the CFCC81428 treatment group than in other treatment groups and the CK group (*p* < 0.05). The H_2_O_2_ contents in the larvae of the BbZ1, Bb01, and CFCC81428 treatment groups were significantly higher than those in the CK group and the remaining treatment groups (*p* < 0.05), with the CFCC81428 treatment group showing the highest content. The PPO activity of larvae in the BbZ1 treatment group was significantly higher than that in other strain treatment groups and the CK group (*p* < 0.05), the activities of larvae in the CFCC81428 and bio-21738 treatment groups were significantly higher than those in the Bb01 and CGMCC3.2055 treatment groups and the CK group (*p* < 0.05), and significant differences in PPO activity were observed among the Bb01 and CGMCC3.2055 treatment groups and the CK group (*p* < 0.05), with the activity decreasing in the order of CGMCC3.2055, Bb01, and CK.

**Figure 4:**
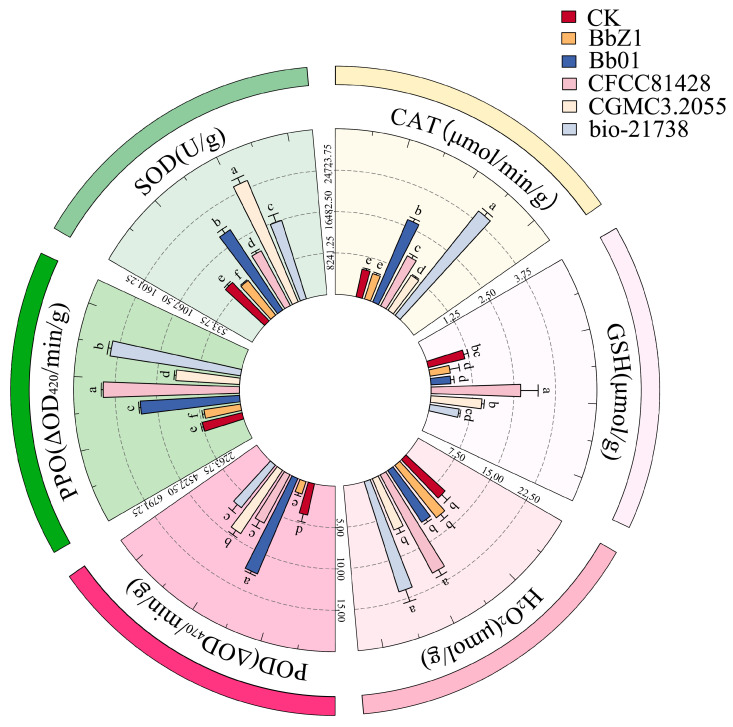
Effects of different *B. bassiana* strains on the main redox system of *D. sylvestrella* larvae at 12 h. Different lowercase letters indicate the statistical differences at the 0.05 significance level (Duncan’s test, *p* < 0.05) in the relevant activities or contents of the larvae of *D. sylvestrella* after treatment with different strains.

At 12 h after the larvae were infected (Figure 4), there were significant differences in the SOD activities of the larvae among each strain treatment group and the CK group (*p* < 0.05), and the activities, in descending order, were CGMCC3.2055, Bb01, bio-21738, CFCC81428, CK, and BbZ1. There was no significant difference in larval CAT activity between the CK group and the BbZ1 treatment group (*p* > 0.05), both of which were significantly lower than those of the other treatment groups, and significant differences were observed in the CAT activity of larvae among the remaining strain treatment groups (*p* < 0.05), with the activities decreasing in the order of bio-21738, Bb01, CFCC81428, and CGMCC3.2055. There was no significant difference in larval CAT activity between the CK group and the BbZ1 treatment group (*p* > 0.05), both of which were significantly lower than those of the other treatment groups, and significant differences were observed in the activity of larvae among the remaining strain treatment groups (*p* < 0.05), with the activities decreasing in the order of bio-21738, Bb01, CFCC81428, and CGMCC3.2055. The POD activity of larvae in the BbZ1 treatment group was significantly lower than that in the CK group and other treatment groups (*p* < 0.05), while the activities of larvae in the remaining strain treatment groups were significantly higher than that in the CK group (*p* < 0.05), and the activity of the CGMCC3.2055 treatment group was significantly lower than that of the Bb01 treatment group and significantly different from that of the other treatment groups (*p* < 0.05). The GSH content of larvae in the CFCC81428 treatment group was significantly higher than that in the CK group and other treatment groups (*p* < 0.05), while there was no significant difference between the CGMCC3.2055 treatment group and the CK group (*p* > 0.05), yet both were significantly higher than those in the BbZ1, Bb01, and bio-21738 treatment groups (*p* < 0.05). There was no significant difference in the H_2_O_2_ content of larvae between the CFCC81428 and bio-21738 treatment groups (*p* > 0.05), but their H_2_O_2_ contents were significantly higher than those in the remaining treatment groups and the CK group (*p* < 0.05). Moreover, significant differences in the PPO activities of the larvae were observed among each strain treatment group and the CK group (*p* < 0.05), with the activities decreasing in the order of CFCC81428, bio-21738, Bb01, CGMCC3.2055, CK, and BbZ1.

**Figure 5:**
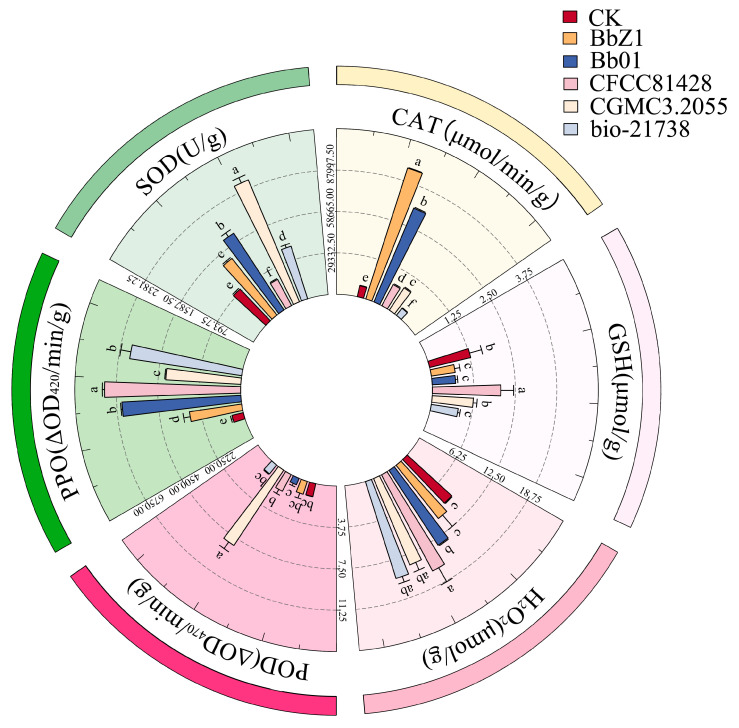
Effects of different *B. bassiana* strains on the main redox system of *D. sylvestrella* larvae at 24 h. Different lowercase letters indicate the statistical differences at the 0.05 significance level (Duncan’s test, *p* < 0.05) in the relevant activities or contents of the larvae of *D. sylvestrella* after treatment with different strains.

At 24 h post-infection (Figure 5), significant differences in the SOD and CAT activities of larvae were observed among each strain treatment group and the CK group (*p* < 0.05), with SOD activities decreasing in the order of CGMCC3.2055, Bb01, BbZ1, bio-21738, CK, and CFCC81428, and CAT activities in the order of BbZ1, Bb01, CGMCC3.2055, CFCC81428, CK, and bio-21738. The POD activity of larvae in the CGMCC3.2055 treatment group was significantly higher than that in the CK group and other treatment groups (*p* < 0.05), while the activity of larvae in the Bb01 treatment group was only significantly lower than that in the CFCC81428 and CGMCC3.2055 treatment groups and showed no significant difference from that in the CK group and other strain treatment groups (*p* > 0.05). The GSH content of larvae in the CFCC81428 treatment group was significantly higher than that in the CK and other treatment groups (*p* < 0.05), while there was no significant difference between the CGMCC3.2055 and CK groups (*p* > 0.05), and the BbZ1, Bb01, and bio-21738 treatment groups had no significant differences among themselves (*p* > 0.05) but were significantly lower than the CK, CGMCC3.2055, and CFCC81428 treatment groups (*p* < 0.05). There was no significant difference in the H_2_O_2_ contents of larvae between the BbZ1 treatment group and the CK group (*p* > 0.05), and both were significantly lower than those of the other strain treatment groups (*p* < 0.05), while the content of larvae in the CFCC81428 treatment group was significantly higher than that in the Bb01 treatment group (*p* < 0.05). No significant difference in larval PPO activities was found between the Bb01 and bio-21738 treatment groups (*p* > 0.05), but they were significantly lower than in CFCC81428 (*p* < 0.05) and higher than in other treatments and CK (*p* < 0.05), with significant differences among CGMCC3.2055, BbZ1, and CK (*p* < 0.05), in descending order.

**Figure 6:**
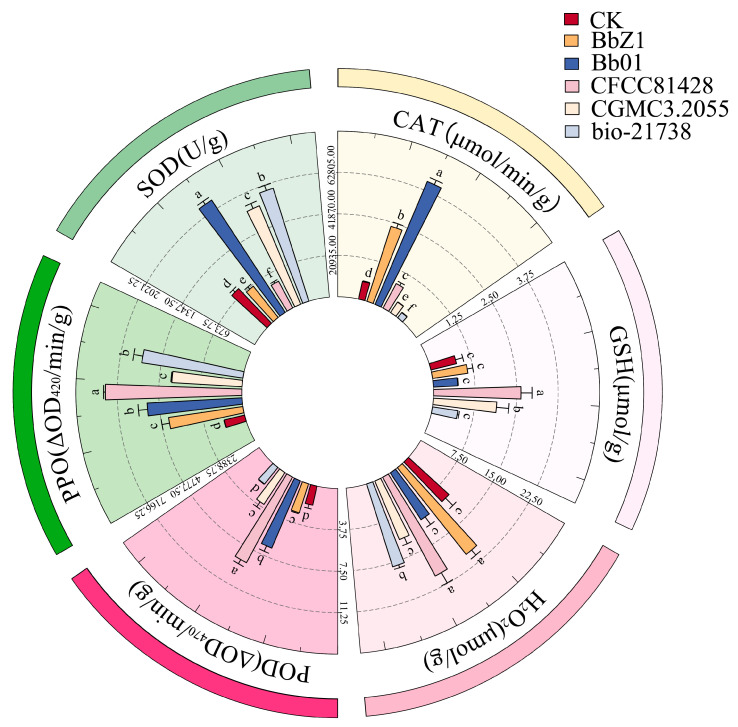
Effects of different *B. bassiana* strains on the main redox system of *D. sylvestrella* larvae at 36 h. Different lowercase letters indicate the statistical differences at the 0.05 significance level (Duncan’s test, *p* < 0.05) in the relevant activities or contents of the larvae of *D. sylvestrella* after treatment with different strains.

At 36 h post-infection (Figure 6), significant differences in the SOD and CAT activities of larvae were observed among each strain treatment group and the CK group (*p* < 0.05), with SOD activities decreasing in the order of Bb01, bio-21738, CGMCC3.2055, CK, BbZ1, and CFCC81428, and CAT activities in the order of Bb01, BbZ1, CFCC81428, CK, CGMCC3.2055, and bio-21738. There was no significant difference in larval POD activities between the bio-21738 and CK groups (*p* > 0.05), and both were significantly lower than other strain groups (*p* < 0.05); no difference between the BbZ1 and CGMCC3.2055 groups’ activities (*p* > 0.05) was found but they were significantly lower than the Bb01 and CFCC81428 groups (*p* < 0.05), with the CFCC81428 group having significantly higher activity than the Bb01 group. The GSH content of larvae in the CFCC81428 treatment group was significantly higher than that in the CGMCC3.2055 treatment group (*p* < 0.05), and both contents were significantly higher than those in the remaining strain treatment groups and the CK group (*p* < 0.05), while there was no significant difference in GSH content among the remaining strain treatment groups and the CK group (*p* > 0.05). The H_2_O_2_ contents of larvae showed no significant difference between the BbZ1 and CFCC81428 treatment groups (*p* > 0.05), both being significantly higher than those of the remaining strain treatments and the CK group (*p* < 0.05), while no significant differences were found among the Bb01 and CGMCC3.2055 treatment groups and the CK group (*p* > 0.05); all were significantly lower than other strain treatments (*p* < 0.05). Larval PPO activity showed no significant difference between the Bb01 and bio-21738 treatment groups (*p* > 0.05), with them being significantly lower than in CFCC81428 (*p* < 0.05) and higher than in other treatments and CK (*p* < 0.05), and there was no difference between the CGMCC3.2055 and BbZ1 groups (*p* > 0.05) but they were significantly higher than in CK (*p* < 0.05).

**Figure 7:**
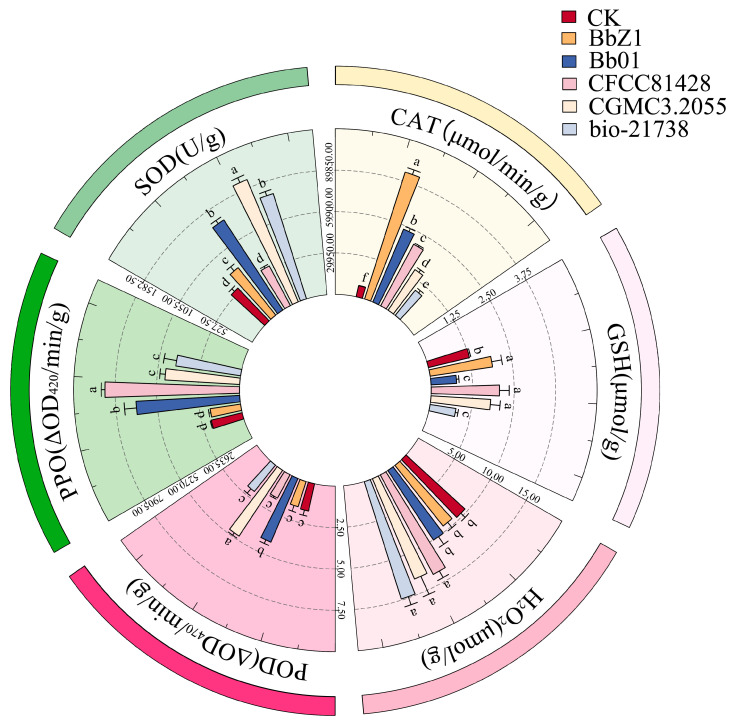
Effects of different *B. bassiana* strains on the main redox system of *D. sylvestrella* larvae at 48 h. Different lowercase letters indicate the statistical differences at the 0.05 significance level (Duncan’s test, *p* < 0.05) in the relevant activities or contents of the larvae of *D. sylvestrella* after treatment with different strains.

At 48 h post-infection (Figure 7), CGMCC3.2055-treated larvae had the highest SOD activity, exceeding other strains and CK; CFCC81428 and CK had the lowest SOD activity (*p* < 0.05) with no intergroup difference; and Bb01 and bio-21738 had lower SOD activity than CGMCC3.2055 (*p* < 0.05). The CAT activities of larvae in each strain treatment group and the CK group showed significant differences (*p* < 0.05), decreasing in the order of BbZ1, Bb01, CFCC81428, CGMCC3.2055, bio-21738, and CK. The CGMCC3.2055-treated larvae had significantly higher POD activity than Bb01-treated ones, with both exceeding other strain treatments and CK (*p* < 0.05), while POD activity did not differ significantly among BbZ1, CFCC81428, bio-21738 treatments and CK (*p* > 0.05). There was no significant difference in the GSH contents of larvae among the BbZ1, CFCC81428, and CGMCC3.2055 treatment groups (*p* > 0.05), but all were significantly higher than those in the CK group and the remaining strain treatment groups (*p* < 0.05). The H_2_O_2_ contents of larvae showed no significant difference among the CFCC81428, CGMCC3.2055, and bio-21738 treatment groups (*p* > 0.05), but all were significantly higher than those in the BbZ1 and Bb01 treatment groups and the CK group (*p* < 0.05). The PPO activity of larvae in the Bb01 treatment group was significantly lower than that in the CFCC81428 group (*p* < 0.05) but higher than in other strain groups and the CK group (*p* < 0.05), the PPO activity did not differ significantly between the CGMCC3.2055 and bio-21738 groups (*p* > 0.05) but was higher than in the CK and BbZ1 groups (*p* < 0.05), and there was no significant difference in PPO activity between the CK and BbZ1 groups (*p* > 0.05).

#### 3.2.3. Effects of *B. bassiana* on the Detoxification Enzymes of *D. sylvestrella* Larvae

**Figure 8:**
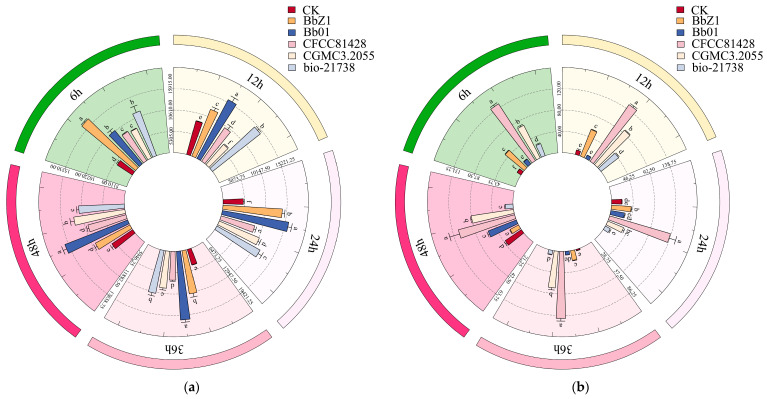
Effects of different *B. bassiana* strains on the detoxifying enzymes of *D. sylvestrella* larvae. (**a**) GST relative activity (nmol/min/g); (**b**) CarE relative activity (ΔOD_450_/min/g). Different lowercase letters indicate the statistical differences at the 0.05 significance level (Duncan’s test, *p* < 0.05) in the relevant activities of the larvae of *D. sylvestrella* at the same time after being treated with different strains.

As depicted in Figure 8, the infection of *D. sylvestrella* larvae by various strains induced distinct alterations in the activities of GST and CarE.

At 6 h after infection, the GST activity of larvae in each strain treatment group was significantly higher than that in the CK group (*p* < 0.05), with the activity in the Bb01 and bio-21738 treatment groups being significantly lower than that in the BbZ1 treatment group (*p* < 0.05) but significantly higher than that in the remaining strain treatment groups (*p* < 0.05). There were significant differences in the CarE activity of larvae among all strain treatment groups and the CK group (*p* < 0.05), and the activity decreased in the order of CFCC81428, CGMCC3.2055, BbZ1, bio-21738, Bb01, and CK.

At 12 h after infection, there were significant differences in the GST activity of larvae among all strain treatment groups and the CK group (*p* < 0.05), with the activity decreasing in the order of Bb01, bio-21738, BbZ1, CFCC81428, CK, and CGMCC3.2055. Regarding CarE activity, only the larvae in the Bb01 treatment group showed no significant difference from the CK group (*p* > 0.05), while the activity in the remaining strain treatment groups was significantly higher than that in the CK group (*p* < 0.05), and there were also significant differences among these treatment groups (*p* < 0.05), decreasing in the order of CFCC81428, CGMCC3.2055, BbZ1, bio-21738, and Bb01.

At 24 h post-infection, GST activity differed significantly among all strain treatments and the CK group (*p* < 0.05), decreasing in the order of Bb01, BbZ1, bio-21738, CGMCC3.2055, CFCC81428, and CK. Meanwhile, for CarE, the CFCC81428 treatment had significantly higher activity than others and the CK group (*p* < 0.05); the BbZ1 treatment had higher activity than the CK, Bb01, and bio-21738 treatments (*p* < 0.05) but no difference from CGMCC3.2055 (*p* > 0.05); the CGMCC3.2055 treatment had higher activity than the CK and bio-21738 treatments (*p* < 0.05) but no difference from Bb01 (*p* > 0.05); and the Bb01 treatment had higher activity than bio-21738 (*p* < 0.05) but no difference from the CK group (*p* > 0.05).

At 36 h post-infection, all strain treatments had significantly higher larval GST activity than the CK group (*p* < 0.05), the BbZ1 and bio-21738 treatments showed no difference in GST activities (*p* > 0.05) but these were lower than Bb01 (*p* < 0.05) and higher than other treatments (*p* < 0.05), and CGMCC3.2055 treatment had significantly higher GST activity than the CFCC81428 treatment (*p* < 0.05). The CGMCC3.2055 treatment had lower CarE activity than CFCC81428 (*p* < 0.05) but higher than other treatments and CK (*p* < 0.05), the BbZ1 treatment had higher CarE activity than Bb01 and bio-21738 treatments and CK (*p* < 0.05), and there was no significant difference in CarE activity among the Bb01 treatment, CK, and bio-21738 treatment groups (*p* > 0.05).

At 48 h post-infection, the CGMCC3.2055 treatment had lower GST activity than Bb01 (*p* < 0.05) but higher than other treatments and CK (*p* < 0.05), the bio-21738 treatment had higher GST activity than the BbZ1 and CFCC81428 treatments and CK (*p* < 0.05), and the BbZ1 and CFCC81428 treatments had similar GST activity (*p* > 0.05), both higher than CK (*p* < 0.05). Significant differences in larval CarE activity existed among the Bb01, CFCC81428, and CGMCC3.2055 treatments and CK (*p* < 0.05)—ranked as CFCC81428, CGMCC3.2055, Bb01, and CK in descending order—while the BbZ1 and bio-21738 treatments had significantly lower CarE activity than CK (*p* < 0.05), with no inter-treatment difference (*p* > 0.05).

### 3.3. Microscopic Structure Observation of D. sylvestrella Larvae Infected by B. bassiana

**Figure 9:**
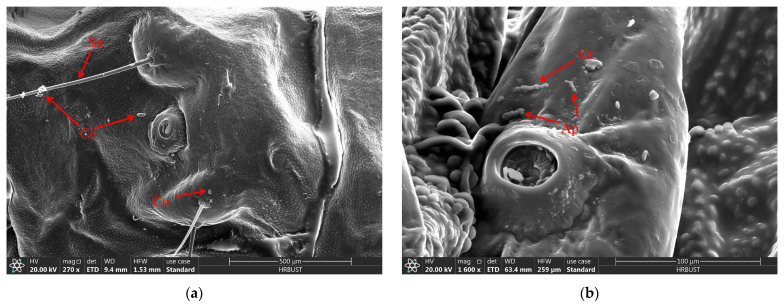

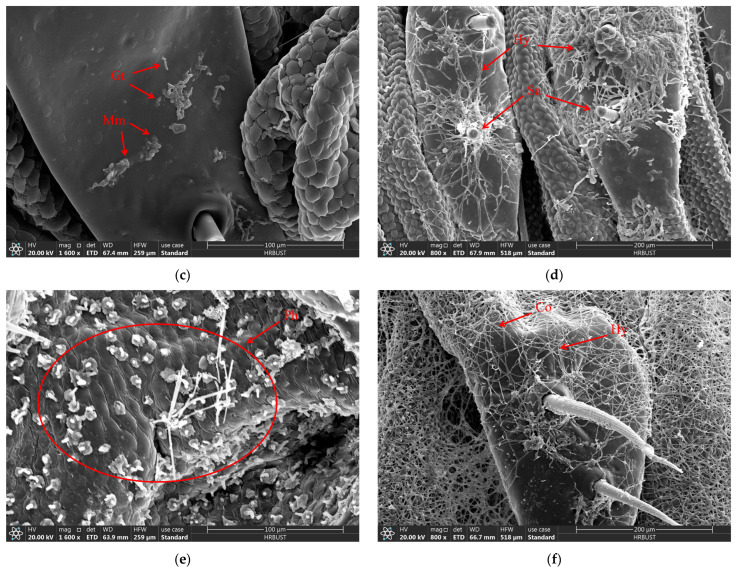
The growth process of *B. bassiana* on the body surface of *D. sylvestrella* larvae. (**a**) At 8 h post-infection; (**b**) 16 h post-infection; (**c**) 24 h post-infection; (**d**) 48 h post-infection; (**e**) 72 h post-infection; (**f**) 84 h post-infection. Conidia (Co); seta (Se); appressorium (Ap); germ tube (Gt); mucilaginous matrix (Mm); hypha (Hy); setal alveolus (Sa); penetrant hyphae (Ph).

When the larvae of *D. sylvestrella* were infected with the strain CGMCC3.2055 for 8 h, the conidia (Co) adhered to the epidermis and setae (Se), and tiny bud-like protrusions appeared on individual conidia (Figure 9a). When infected for 16 h, germ tubes (Gts) formed, grew along the epidermis, and the ends and other parts of some germ tubes expanded, resulting in the formation of appressoria (Ap) (Figure 9b). At 24 h post-infection, numerous Gts formed and penetrated the cuticle, with a mucous layer (Mm) visible at the contact site between the germ tube tips and the larval cuticle, indicative of tissue dissolution around the cuticle by the mycelium (Figure 9c). At 48 h post-infection, numerous hyphae (Hy) intertwined around the setal alveoli (Sa), grew along the epidermis, and penetrated it (Figure 9d). However, at 72 h post-infection, it was found that a large number of penetrating hyphae (Ph) had already emerged from the surface of the insect body (Figure 9e). At 84 h post-infection, Hy had spread across the body surface and formed a reticular structure, and new Co began to grow (Figure 9f).

## 4. Discussion

Entomopathogenic fungi, which can invade diverse insect hosts in nature and are eco-friendly pest control alternatives to chemical insecticides, prompt insects to counter invading pathogens mainly through behavioral avoidance, physical barriers like cuticles and innate immune defenses including cellular and humoral immunity [19]. However, the antibacterial activity within insects can also be achieved by generating ROS [20]. When insects are confronted with the invasion of foreign substances, an excessive amount of ROS in their bodies will disrupt the balance between the oxidative and antioxidant mechanisms within cells and organisms, and an oxidative stress response will be generated in insects to avert the harmful effects of excessive ROS on DNA, lipids, proteins, and other biomolecules [11,21,22]. Among them, ROS mainly includes superoxide anion (O^2−^), H_2_O_2_, hydroxyl radical (OH^−^), ozone (O_3_) and singlet oxygen (^1^O_2_) [23]. In their study on the oxidative damage of *Arma chinensis* nymphs under Cd exposure, Sun Guotong et al. [24] found that the contents of MDA and H_2_O_2_ in the nymphs increased significantly, and the nucleus, nuclear membrane, mitochondria and other structures in the midgut tissue were also damaged. Our study showed that T-AOC in *D. sylvestrella* larvae was inhibited within 48 h post-infection by strains CGMCC3.2055 and CFCC81428, while in other strain-treated groups, it increased only at 6 or 12 h and remained lower than the CK group at other time points, indicating that excess ROS could not be efficiently cleared post-infection. As a key marker of the degree of oxidative stress, MDA effectively reflects the degree of lipid peroxidation in the organism [25]. Our study found that the MDA content in *D. sylvestrella* larvae increased significantly after infection by different *B. bassiana* strains, with the relative MDA content in larvae treated with strain CGMCC3.2055 being significantly higher than that in other strain-treated groups at 6 h, 12 h, and 24 h post-infection and only lower than that in the groups treated with strains bio-21738 or CFCC81428 at 36 h and 48 h; this is in accordance with the mortality of *D. sylvestrella* larvae caused by different strains and indirectly indicates that *D. sylvestrella* larvae have weak total antioxidant capacity against *B. bassiana*, especially strain CGMCC3.2055, impairing ROS scavenging, leading to an excessive accumulation of MDA and ROS, and ultimately affecting larval viability. Mao Genlin et al. [26] demonstrated that after inducing oxidative stress in the gut of *Spodoptera litura* with Bruceine D, the excessive accumulation of ROS and MDA led to cell apoptosis and damaged the midgut tissue. Therefore, it can be confirmed that after the larvae of *D. sylvestrella* are infected by *B. bassiana*, the excessive accumulation of ROS and MDA in their bodies will affect their own viability.

When insects are confronted with the invasion of foreign substances, the SOD in their bodies will catalyze the disproportionation reaction of O_2_⁻ to scavenge O_2_⁻, but toxic H_2_O_2_ will be produced [27,28]. As the main antioxidant-related enzymes, POD and CAT can counteract the overexpression of H_2_O_2_ and maintain normal functions [29,30]. As an antioxidant, GSH can directly undergo reduction reactions with ROS such as H_2_O_2_ [31], and it also exerts a protective effect against H_2_O_2_-induced cellular oxidative stress [32]. Although H_2_O_2_ can serve as a signaling molecule and play an important role in responses to stress, programmed cell death, and the regulation of growth and development, an excessive amount of H_2_O_2_ will not only lead to cellular damage and dysfunction but also exert other toxic effects [29,33]. In our study, the oxidative stress response of *D. sylvestrella* larvae to *B. bassiana* was obvious at 12 h post-infection. Except for the BbZ1 group, SOD, CAT and POD activities were upregulated in other strain-treated groups, though H_2_O_2_ levels increased in some groups. When infected with the highly pathogenic strain CGMCC3.2055 from 12 to 48 h post-infection, larval SOD activity rose continuously, reacting with O_2_^−^ to produce H_2_O_2_. At 12 h post-infection, increased CAT and POD activities countered H_2_O_2_ overexpression. At 24 h post-infection, these enzymes could not fully offset H_2_O_2_, causing accumulation. At 36 h post-infection, GSH levels surged, compensating for reduced CAT activity and working with POD to restore H_2_O_2_ levels. However, at 48 h post-infection, upregulated GSH, CAT, and POD could not fully counter H_2_O_2_ overaccumulation. Therefore, we conclude that during *D. sylvestrella* larvae infection by strain CGMCC3.2055, ROS are mainly regulated by SOD, CAT, and POD. Antioxidant enzymes are crucial for ROS elimination in insect–fungus pathogenesis. Karthi et al. [34] reported that *S. litura* treated with *Aspergillus flavus* had significantly higher SOD activity than their control group. Chaurasia et al. [35] found that CAT activity decreased in the midgut and fat body of *Periplaneta americana* after infection by different entomopathogenic fungi, likely due to free radical production imbalance suppressing innate defenses or CAT’s inefficiency against fungal-induced oxidative stress. However, we found that the activities of CAT and SOD in *D. sylvestrella* larvae infected with strain Bb01 increased significantly from 6 to 48 h post-infection. Zhou et al. [36] found that in third-instar *Sogatella furcifera* nymphs, thiamethoxam initially upregulated the activities of POD and SOD, whereas buprofezin had the opposite effect, and the activities of CAT varied across different concentrations of these two insecticides. All these findings indicate that the stress responses in insects can vary depending on different foreign harmful substances. In our study, it was also found that within different time periods after infection, the contents of GSH and H_2_O_2_ in *D. sylvestrella* larvae of the treatment group with strain CFCC81428 were generally higher than those in the other treatment groups, and the T-AOC activity in the larvae was significantly inhibited, yet the larval mortality was not higher than that in the treatment groups with strains CGMCC3.2055 and Bb01. Seo et al. [37] found that by evaluating the tolerance of the CAT and glutathione system to H_2_O_2_, an increase in the content of GSH can enhance the tolerance to lethal oxidative damage. Therefore, we believe that the larvae of *D. sylvestrella* have tolerance to the oxidative damage caused by the infection of strain CFCC81428.

When confronted with foreign substance invasion, the detoxification system in insects operates in three stages, with GST and CarE participating in the detoxification metabolism of the first and second stages, respectively [38]. As one of the innate immune mechanisms in insects, PPO, which is often involved in cellular and humoral immunity, is activated by serine proteases to induce a melanization reaction around invading pathogens for resisting infection [39,40]. Moreover, PPO has a detoxifying effect on the ROS released during the intake of pro-oxidative allelochemicals [41]. Some studies have shown that when the RNA interference (RNAi) technique is used to inhibit the expression of the PPO gene in *Acyrthosiphon pisum*, the mortality rate of *A. pisum* infected by *B. bassiana* increases significantly [42]. This demonstrates the importance of PPO in the immune defense of insects. In this study, except for the group treated with strain BbZ1, the PPO activity of *D. sylvestrella* larvae in the other strain-treated groups continuously increased from 6 to 48 h post-infection to resist *B. bassiana* infection, the GST activity in the larvae significantly increased after infection by various *B. bassiana* strains with a decrease only observed at 12 h post-infection by strain CGMCC3.2055, and the CarE activity in the larvae of the treatment groups with strains BbZ1, CFCC81428, and CGMCC3.2055 also continuously increased from 6 to 36 h post-infection. This may be due to the significant differences in the virulence of *B. bassiana* towards different host pests [43]. Moreover, the expression patterns of insects vary when they are confronted with different strains of *B. bassiana*, leading to differences in their own immune defense mechanisms [10]. Studies have shown that the same strain of *B. bassiana* PfBb exhibits different lethal effects and detoxifying enzyme activities on larvae of *Spodoptera frugiperda* at different instar stages [44]. Therefore, although the larvae of *D. sylvestrella* exhibit immune defense responses against different strains of *B. bassiana*, the expression levels of CarE, GST, and PPO in the larvae vary, thereby influencing the main immune responses of the larvae to each strain and the detoxification effects at different stages.

Entomopathogenic fungi with a broad host spectrum have conidia adhere to the insect cuticle, germinate under favorable conditions, penetrate the cuticle via mechanical pressure and enzyme secretion, transform into blastospores in the hemocoel to colonize, evade the host immune system, secrete toxins to kill the host, and finally form conidia on the cadaver [7,45]. This is consistent with the infection steps of *B. bassiana* CGMCC3.2055 on *D. sylvestrella* larvae. Entomopathogenic fungi typically penetrate the insect cuticle via infection pegs generated by appressoria or the direct entry of germ tubes during host invasion [46] (pp. 268–270). In response, insect epiderma [7,45] cells generate antimicrobial peptides and ROS to combat the fungal invasion [19]. Meanwhile, an imbalance of ROS within the insect host triggers oxidative stress responses [11]. Since SEM can only be used to observe the process of the strain infecting the larval epidermis, and the oxidative stress response can only focus on the immune defense response of the larval body against the strain, we combined the oxidative stress response of the larvae of *D. sylvestrella* to the strain CGMCC3.2055 with the process of the strain infecting the larval epidermis. By jointly discussing the infection process and the oxidative stress response in the larvae, we found that when the larvae were infected for 6 h, the oxidative stress response in their bodies was not obvious, likely due to the fact that a large number of Co have not formed invasive germ tubes at this stage. At 8 h, Co adhered to the larval epidermis with bud-like protrusions for epidermal invasion. At 12 h, larval SOD, CAT, and POD regulated ROS and cleared excess H_2_O_2_, which was likely caused by the invasion of the larval epidermis by Gts. At 16 h, it can be clearly observed that the Gts have grown along the epidermis and some of the Gts have produced Ap to invade the epidermis. At 24 h, a large number of Gts were clearly observed to penetrate the epidermis, accompanied by the dissolution of the tissues around the epidermis by hyphae, and at this time, the oxidative stress response in the larvae was relatively pronounced, with CAT and POD being unable to eliminate the excess H_2_O_2_. At 36 h, larval GSH started to regulate H_2_O_2_. At 48 h, Hy coiled around and penetrated the larval epidermis at permeable sites like Sa, triggering a pronounced oxidative stress response in the larvae. At 72 h, the larvae were colonized by the strain, with numerous Ph emerging from the larval body surface. At 84 h, hyphae had formed a reticular structure, and new conidia began to grow. When exploring mature *Carposina sasakii* larvae infected by *B. bassiana* strain TST05, it was found that conidia attach to the cuticle, form germ tubes, penetrate the cuticle, trigger melanization in the cuticle and hemolymph, and ultimately infect internal organs [47]. Therefore, we can preliminarily determine the ultrastructure of the cuticle of *D. sylvestrella* larvae infected by *B. bassiana* CGMCC3.2055.

## 5. Conclusions

**Figure 10:**
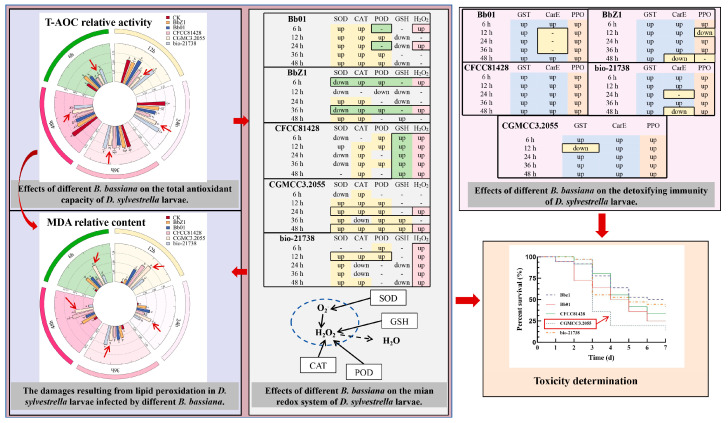
Related conclusions on screening highly pathogenic strains based on the redox reactions, detoxification immunity, and virulence determination of *D. sylvestrella* larvae after being infected by *B. bassiana*. In the figures of T-AOC and MDA, the red arrows indicate the activity or content of larvae under the treatment of CGMCC3.2055.

As shown in Figure 10, in this study, the virulence of five *B. bassiana* strains (Bb01, CGMCC3.2055, BbZ1, bio-21738, and CFCC81428) against the fourth-instar larvae of *D. sylvestrella* was determined, and then by combining the contents of T-AOC and MDA in the larvae infected by each strain, it was determined that strain CGMCC3.2055 had high pathogenicity to the larvae. It mainly inhibited the total antioxidant capacity of the larvae. This affected the scavenging of ROS, causing excessive accumulation of MDA and ROS, and thereby influenced the activity of the larvae. On this basis, a further exploration of the impact of each strain on the main redox system of the larvae revealed that in the larvae of the Bb01 treatment group, SOD, CAT, and POD were involved in the redox reaction, and the content of H_2_O_2_ was not affected by GSH but by the activity of POD, resulting in the accumulation of H_2_O_2_ in the larvae at 6 and 24 h post-infection. In the larvae of the BbZ1 treatment group, the content of H_2_O_2_ accumulated significantly only at 6 and 36 h, which was not caused by the dismutation reaction of SOD, and the upregulation of CAT and POD was insufficient to effectively remove the excess H_2_O_2_. In the larvae of the CFCC81428 treatment group, the T-AOC decreased significantly, H_2_O_2_ accumulated greatly within 48 h, and the upregulation of CAT and POD could not effectively eliminate the excess H_2_O_2_, but the content of GSH increased significantly, thus enhancing the larvae’s tolerance to lethal oxidative damage. In the larvae of the CGMCC3.2055 treatment group, SOD, CAT, and POD all participated in the redox reaction, the content of H_2_O_2_ only accumulated significantly at 24 and 48 h, and the upregulation of CAT and POD could not effectively remove the excess H_2_O_2_. In the larvae of the bio-21738 treatment group, SOD underwent dismutation from 12 to 48 h, generating a large amount of H_2_O_2_, and the content of H_2_O_2_ accumulated significantly from 6 to 48 h, while POD was only upregulated at 6 and 12 h and CAT only at 12 and 48 h, so the H_2_O_2_ was not effectively cleared. In this study exploring the detoxification and immunity of the larvae against each strain, it was found that upon infection by five *B. bassiana* strains, *D. sylvestrella* larvae utilize PPO for immune defense via cellular and humoral pathways, with PPO activity upregulating within 48 h in all treatment groups except BbZ1 at 12 and 48 h. Meanwhile, GST and CarE mediate detoxification, as GST activity increased in most groups except in the CGMCC3.2055 group at 12 h post-infection. CarE activity significantly increased within 48 h in the CFCC81428 and CGMCC3.2055 groups, increased in the Bb01 group only at 6 and 48 h, and decreased in the BbZ1 group only at 48 h, with no upregulation in the bio-21,738 group only at 24 and 48 h.

**Figure 11:**
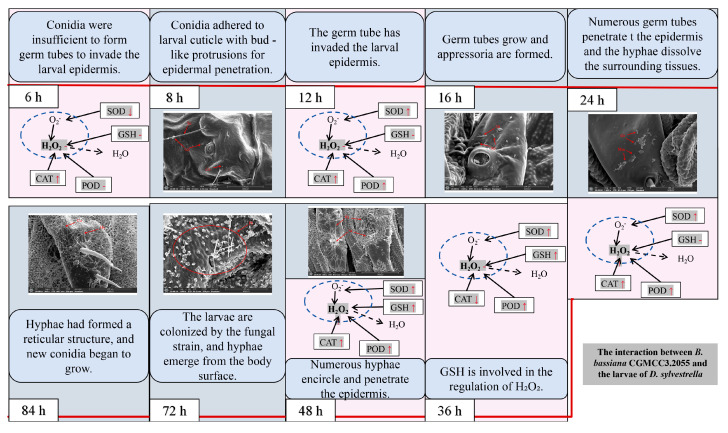
Conclusions related to the infection process of highly pathogenic *B. bassiana* strains on the larvae of *D. sylvestrella*. The red arrows ↑, ↓ and - indicate up-regulation, down-regulation and no significant change in the activity or content of larvae under the treatment of CGMCC3.2055 strain, respectively.

As shown in Figure 11, a combined analysis of the oxidative stress response of *D. sylvestrella* larvae to strain CGMCC3.2055 and the infection process revealed the following: At 6 h, due to the fact that the conidia have not formed germ tubes to invade the larval epidermis, the oxidative stress response in the larvae is not obvious. At 8 h, the conidia adhere and show bud-like protrusions. At 12 h, the invasion of germ tubes triggers the activation of SOD, CAT, and POD for ROS regulation. At 16 h, the germ tubes grow and appressoria are formed. At 24 h, CAT and POD are insufficient to clear excess H_2_O_2_, numerous germ tubes penetrate the epidermis, and the hyphae dissolve the surrounding tissues. At 36 h, GSH is involved in the regulation of H_2_O_2_. At 48 h, a large number of hyphae wind around and penetrate the epidermis at easily permeable parts such as the setal sockets of the larvae, and the oxidative stress response is relatively obvious. At 72 h, the larvae are colonized by the fungal strain, and hyphae emerge from the body surface. At 84 h, hyphae form a reticulum and new conidia start growing. This study provides a theoretical basis for the subsequent development of highly effective biological control agents and in-depth exploration of the pathogenic mechanism of *B. bassiana* against the larvae of *D. sylvestrella*.

## Figures and Tables

**Table 1:** Basic information of tested strains.

Strain No.	Strains	Original Hosts
CGMCC3.2055	*B* *. bassiana*	*Dendrolimus* spp.
CFCC81428	*B. bassiana*	*Monochamus alternatus*
bio-21738	*B. bassiana*	*Dendrolimus punctatus*
Bb01	*B. bassiana*	*Xylotrechus rusticus*
BbZ1	*B. bassiana*	*Hylurgus ligniperda*

**Table 2:** LT_50_ of different *B. bassiana* strains against *D. sylvestrella* larvae.

Strains	Regression Equation	Goodness of Fit Test χ^2^	*p* Value	LT_50_ (d)	95% Confidence Interval
BbZ1	Y = −1.788 + 0.980x	5.914	0.998	6.200	5.116–8.410
Bb01	Y = −1.532 + 1.162x	8.878	0.975	3.739	3.214–4.347
CGMCC3.2055	Y = −2.265 + 1.922x	22.453	0.262	3.248	2.884–3.593
Bio-21738	Y = −2.045 + 1.285x	22.222	0.273	4.911	4.290–5.792
CFCC81428	Y = −2.522 + 1.621x	10.020	0.952	4.737	4.237–5.364

## Data Availability

The original contributions presented in this study are included in the article. Further inquiries can be directed to the corresponding authors.
